# A new galling insect model enhances photosynthetic activity in an obligate holoparasitic plant

**DOI:** 10.1038/s41598-021-92417-3

**Published:** 2021-06-21

**Authors:** Ryo Murakami, Ryo Ushima, Ryoma Sugimoto, Daisuke Tamaoki, Ichirou Karahara, Yuko Hanba, Tatsuya Wakasugi, Tsutomu Tsuchida

**Affiliations:** 1 Graduate School of Science and Engineering for Education, University of Toyama, Toyama, Toyama 930-8555 Japan; 2 Faculty of Science, Academic Assembly, University of Toyama, 3190 Gofuku, Toyama, Toyama 930-8555 Japan; 3 Faculty of Applied Biology, Kyoto Institute of Technology, Matsugasaki, Sakyo-ku, Kyoto, 606-8585 Japan

**Keywords:** Entomology, Evolutionary ecology

## Abstract

Insect-induced galls are microhabitats distinct from the outer environment that support inhabitants by providing improved nutrients, defence against enemies, and other unique features. It is intriguing as to how insects reprogram and modify plant morphogenesis. Because most of the gall systems are formed on trees, it is difficult to maintain them in laboratories and to comprehend the mechanisms operative in them through experimental manipulations. Herein, we propose a new model insect, *Smicronyx madaranus*, for studying the mechanisms of gall formation. This weevil forms spherical galls on the shoots of *Cuscuta campestris*, an obligate parasitic plant. We established a stable system for breeding and maintaining this ecologically intriguing insect in the laboratory, and succeeded in detailed analyses of the gall-forming behaviour, gall formation process, and histochemical and physiological features. Parasitic *C. campestris* depends on host plants for its nutrients, and usually shows low chlorophyll content and photosynthetic activity. We demonstrate that *S. madaranus-*induced galls have significantly increased CO_2_ absorbance. Moreover, chloroplasts and starch accumulated in gall tissues at locations inhabited by the weevil larvae. These results suggest that the gall-inducing weevils enhance the photosynthetic activity in *C. campestris*, and modify the plant tissue to a nutrient-rich shelter for them.

## Introduction

Plant galls are abnormally growing tissues resulting from complex cross-kingdom interactions. Galls are microhabitats distinct from the outer environment that support specialist inhabitants by improving nutritional compositions and their adaptive structures for galler growth, defence against enemies, and mitigating environmental stress^1,2^. The plant gall functions as an “extended phenotype” for inducers^3^.

Although various organisms, including viruses, bacteria, fungi, nematodes, and mites, have the ability to induce galls in plants^1^, insect-induced galls are distinct, being more complex, highly organized, and diverse^4,5^. Hence, it is intriguing as to how such insects reprogram and modify plant morphogenesis at the molecular level. However, because many of the insect-induced galls are formed on woody plants^6,7^, it is difficult to maintain them in laboratories and to identify the factors involved in the mechanisms operative in such galls through experimental manipulations. The Hessian fly (*Mayetiola destructor*) has become a representative model galling insect that can be reared on a non-woody plant, wheat^8^. However, the gall induced by the larvae inhabiting the sheath does not show remarkable morphological changes. Thus, studies with the Hessian fly would not explain all the mechanisms underlying the formation of different and highly organized insect-induced galls.

**Figure 1:**
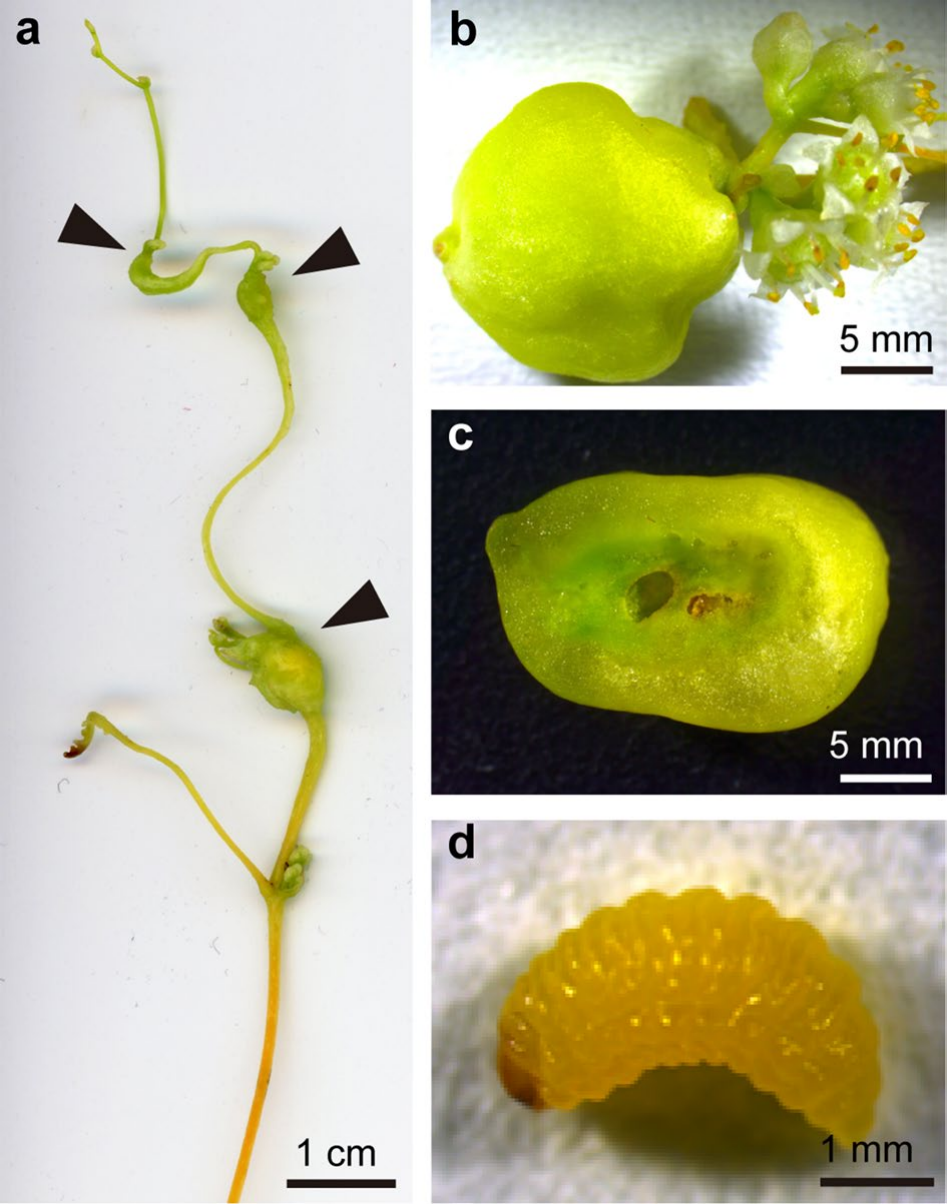
*Smicronyx madaranus*-induced galls on *Cuscuta campestris*. (**a**) Galls formed at branching points on a shoot, (**b**) a gall formed at the base of an inflorescence. (**c**) transverse section of a mature gall, (**d**) *S. madaranus* larva inhabiting a mature gall. Arrow head, gall.

In this paper, we propose a new model insect, *Smicronyx madaranus* Kono, for studying the mechanisms underlying gall formation. This weevil species was first reported in Iwate, Japan^9^. It is distributed in Japan (main island and Kyushu district), Korea, and the Russian Far East^10^. The major host of this weevil is the obligate parasitic plant, field dodder *Cuscuta campestris* Yuncker (synonym: *C. pentagona* Engelm). *Cuscuta australis* R.Br. and *C. chinensis* Lam. were also reported as host plants^10^, but the status of the latter as a host is disputable, according to Hayakawa et al.^11^, who showed little evidence for it to be a host based on comprehensive field and specimen surveys. *Smicronyx madaranus* forms baccate spherical galls coloured pale orange to green on the shoots of the field dodder, *C. campestris* (Fig. 1). We established a stable system for breeding and maintaining this ecologically intriguing gall-forming insect in the laboratory, and succeeded in detailed analyses of the gall-forming behaviour, gall formation process, and histochemical and physiological features. We demonstrate that *S. madaranus*-induced gall on the obligate parasitic plant significantly increased the photosynthetic activity and offered a nutrient rich shelter to the weevil.

## Results

### Location of the gall formation

We observed the location of the weevil-induced galls on the dodder in the field. Galls of the weevils were observed on the nodes (branching points) of the shoots of *C. campestris* but not on internodes (stem proper) (Fig. 1a). Some galls were observed at the basal nodes of the inflorescences (Fig. 1b). Small galls were found on the nodes closer to the shoot apex, whereas larger galls were found on the nodes closer to the shoot base. Galls were also observed at the same locations on *C. campestris* in the laboratory (Fig. S1).

### Egg-laying behaviour of the weevils

Time-lapse photography confirmed the egg-laying behaviour of the weevil; an adult female deposited an egg into the node at the tip of the dodder shoot after boring a hole with its long rostrum (Movie S1). This behaviour was observed four times during the 5-day experimental period. The plant-boring behaviour was observed at various points (Movie S2), but the egg-laying behaviour was only observed at the nodes.

### Gall formation schedule and escape timing of the weevil

**Figure 2:**
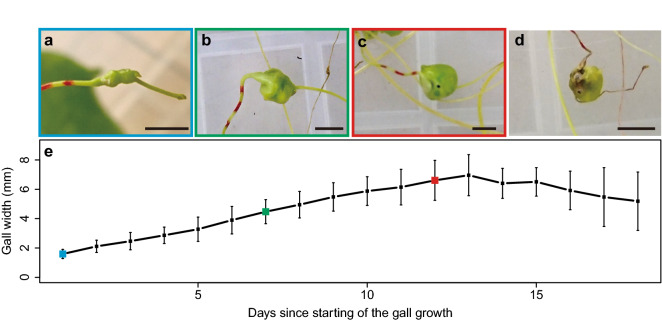
Growth of galls and changes in their appearance. (**a**) The earliest gall at the 1st day, (**b**) growing gall at 7th day, (**c**) 12th day gall just after the escape of weevil, and (**d**) withered gall at 21st day. Bars, 5 mm. (**e**) Changes in the gall size over time. Means ± standard deviations are shown. Sample size is 10. Colours around the images of (**a**), (**b**) and (**c**) correspond to the colours in the graph.

We recognized small galls (1.5–2 mm in width) 3 to 7 days after the introduction of weevils (Fig. 2a). This day is referred to as the first day of gall-forming in this paper. Thereafter, the galls grew gradually and became larger (Fig. 2b), and the last-instar larvae of weevil made small holes in the galls and escaped from the galls (Fig. 2c) from day nine to 15 (mean ± SD and median values were 12.4 ± 2.17 days and 12 day, respectively). The gall size increased during the period of larval growth, reaching a peak (mean ± SD of the width was 6.96 ± 1.40 mm; mean ± SD of the volume was 192.20 ± 84.07 mm^3^) on the 13th day that corresponded to the time of weevil escaping, and decreased after the escaping (Fig. 2e). The galls from which the weevil escaped eventually withered (Fig. 2d). The stem width of the nodes before gall formation was 0.944 ± 0.130 mm (mean ± SD). The volume of the nodal regions before gall formation was 4.96 ± 1.34 mm^3^ (mean ± SD), when its length was the same as that of the mature gall. This suggests that the tissue volume increased by approximately 38.75 times during gall formation.

### Gross and histological appearance of the gall

**Figure 3:**
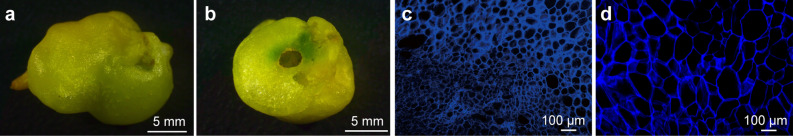
Gross and histological appearance of the weevil-induced gall. Gall in the mature stage. (**a**) whole gall, (**b**) transverse sections of the gall, (**c**) fluorescent image of inner tissue, and (**d**) outer tissue stained with Calcofluor.

Shoots of the field dodder are usually yellow-orange in colour, whereas the weevil-induced galls were green (Fig. 1a). The cross section of the mature gall showed a cavity in the centre of the gall where the larva of weevil inhabited (Fig. 1c,d). In the cavity, the larva was observed to vigorously feed upon the inside tissues (Movie S3). Tissues around the cavity were dark green; whereas the outer tissue was light green (Fig. 1c). Differences between the inner and outer layers were also found in the cell size at mature stages of the galls (Fig. 3a–d). The cells around the cavity inhabited by the larva were small (Fig. 3c). The outer layer consisted of enlarged cells (Fig. 3d).

**Figure 4:**
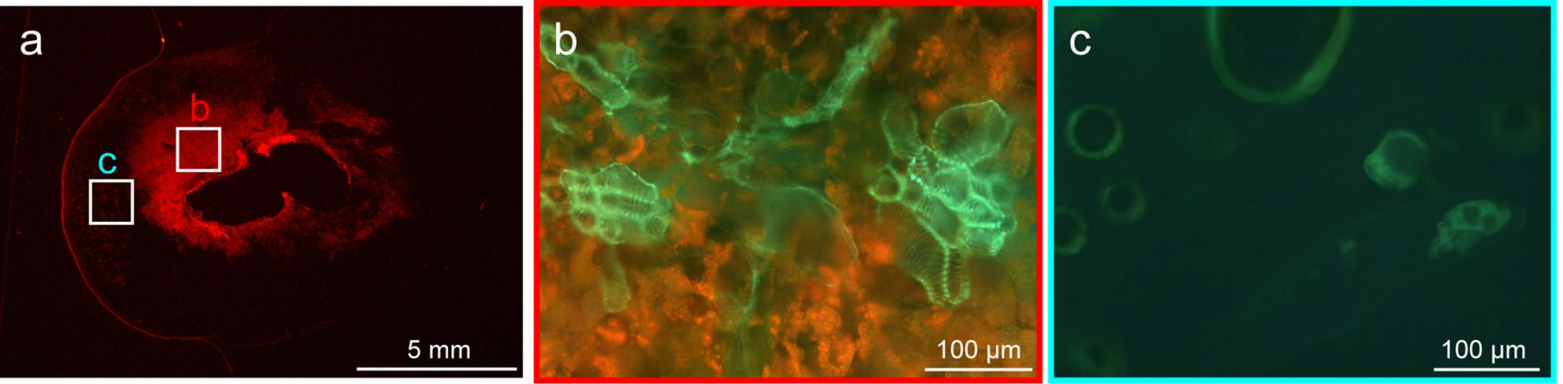
Internal structure of the mature gall. Autofluorescence of chlorophylls (red) and lignin (green). (**a**) sagittal section of the whole gall, (**b**) magnified image of the inner areas, and (**c**) magnified image of the outer areas.

Fluorescence microscopy revealed strong chlorophyll autofluorescence signals in the inner layer (Fig. 4a,b). Lignin autofluorescence was also detected in the vascular bundles in the inner layer (Fig. 4b). In contrast, signals for chlorophylls and vascular bundles were rarely detected in the outer layer (Fig. 4c). In the nodal region, the colour was slightly green (Fig. S2a,b). In the nodal region, very weak signals for chlorophylls and chloroplasts were detected (Fig. S2c,e), whereas few signals were detected at the internodes (Fig. S2c,d).

### Concentration of chlorophylls, photosynthetic activity, and starch accumulation in the gall and shoots

**Figure 5:**
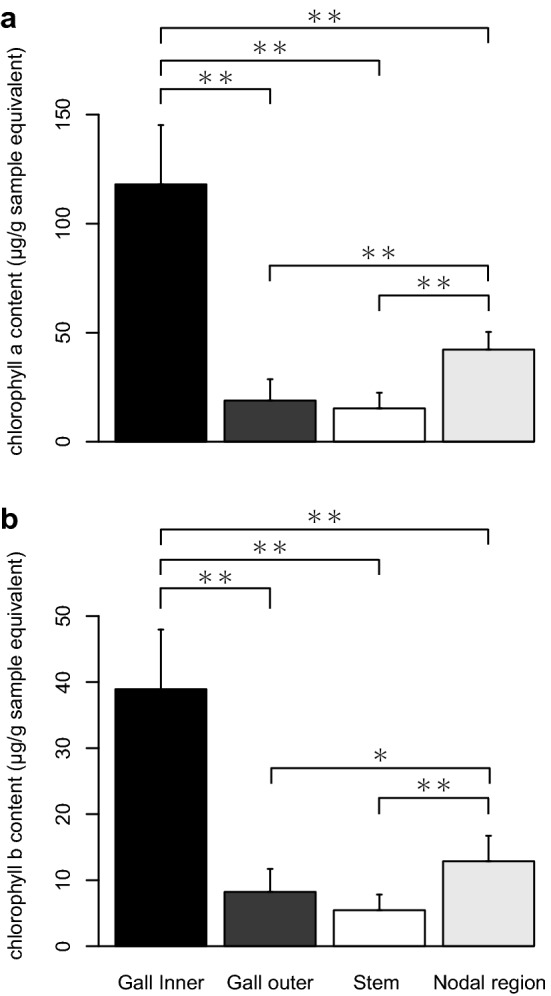
Chlorophyll content in *Cuscuta campestris*. (**a**) Chlolrophyll *a* and (**b**) chlorophyll *b* contents. The values are shown as means ± SD. Asterisk indicates statistically significant difference (*P < 0.05; **P < 0.01; Wilcoxon rank sum test with Bonferroni correction).

Spectrophotometric determination showed that the inner layer of galls contained a significantly larger amount of chlorophyll (Chl) *a* than the other parts (Fig. 5a). The concentration of Chl *a* in the intact tissue around the node was significantly higher than in the outer layer of gall and stem. The concentrations of Chl *b* were relatively lower than those of Chl *a* in all parts of the dodder (Fig. 5b). The Chl *b*/*a* ratio was the same in each part of the dodder. The concentrations of Chl *a* and *b* in the entire gall, including both inner and outer layers, were 68.48 ± 13.98 and 23.58 ± 5.52 µg/g sample equivalent, respectively. In the nodal regions, Chl *a* and *b* concentrations were 42.26 ± 8.08 and 12.86 ± 3.87 µg/g sample equivalent, respectively. Hence, the mean concentrations of the chlorophylls in the galls were approximately 1.6- and 1.8-times higher than that in the nodal regions.

**Figure 6:**
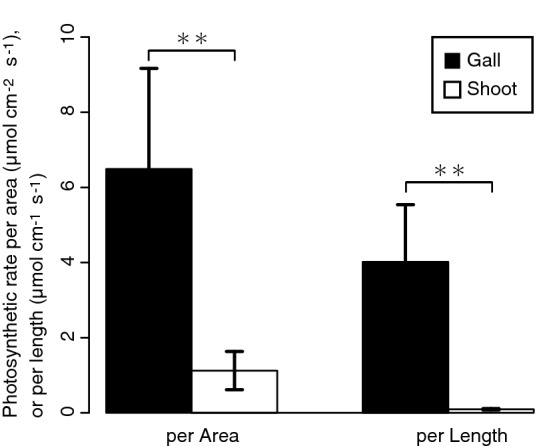
Photosynthetic rates in the gall and shoot. The values are shown as means ± SD. Asterisk indicates statistically significant difference (**P < 0.01; Welch’s t test).

CO_2_ absorption was more active in galls than in shoots (Fig. 6). The mean CO_2_ absorption rates per area and per length were approximately 5.8- and 42.7-times higher in galls than in the shoots, respectively (Fig. 6).

**Figure 7:**
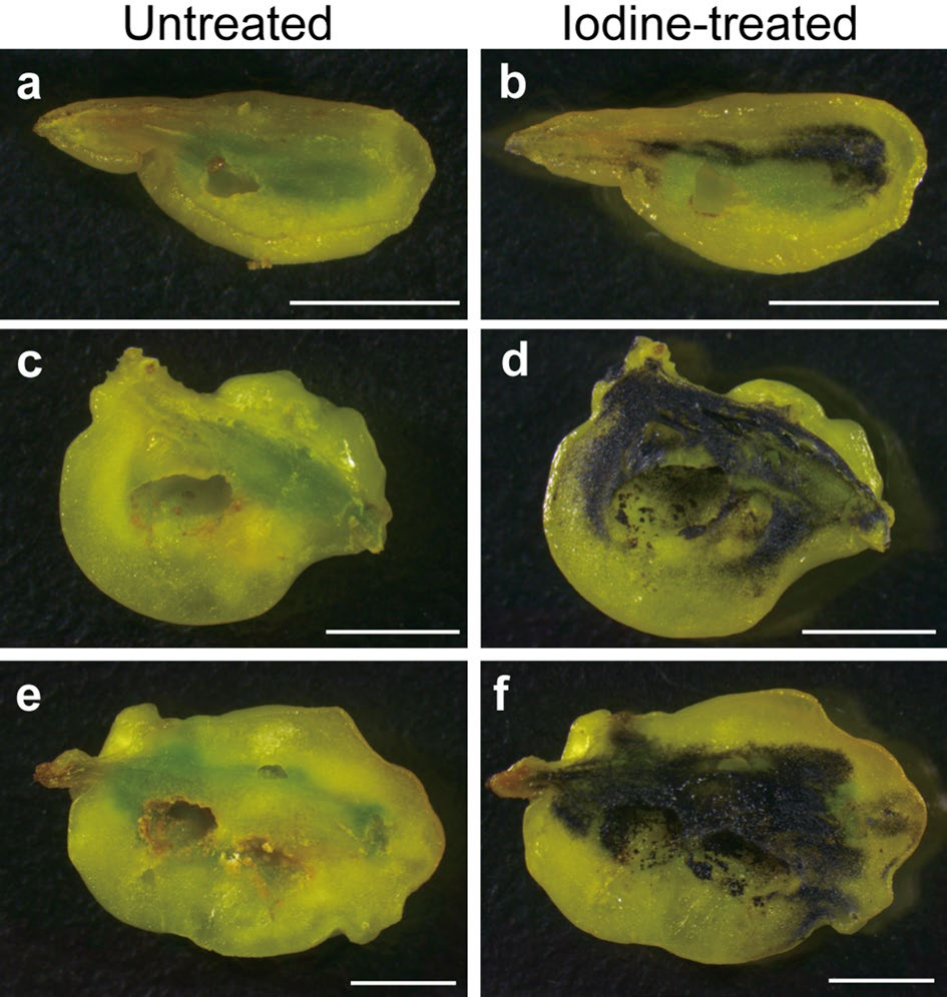
Distribution of starch in the gall. (**a**,**b**) Sagittal section of the early mid stage, (**c**,**d**) late mid stage, and (**e**,**f**) mature stage of the galls. In (**b**,**d**,**f**), starch was detected as blue-black colour upon staining with Lugol’s iodine solution. Bars, 5 mm.

**Figure 8:**
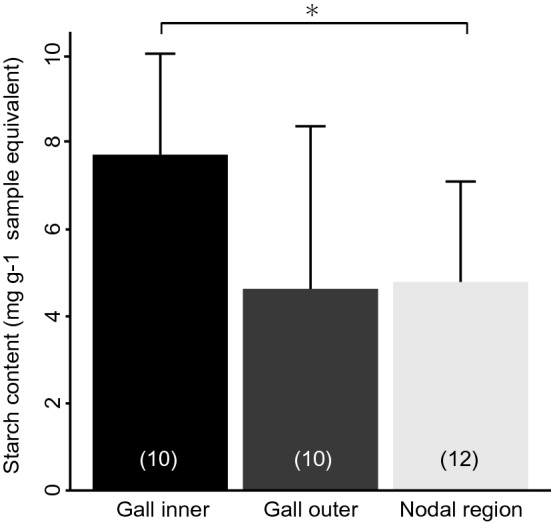
Starch content in *Cuscuta campestris*. The values are shown as means ± SD. Asterisk indicates statistically significant differences (*P < 0.05; Wilcoxon rank sum test with Bonferroni correction). Sample size is given in parentheses.

The distribution of starch was consistent with that of chlorophylls shown in dark green colour on the dodder (Fig. 7a–f). In the shoots, a small amount of starch was detected around the nodes, but not in the rest of the portions (Fig. S3a–f). In the galls, starch was found around the cavity inhabited by the weevil larva, and the amount of starch increased with the growth of galls (Fig. 7b,d,f). Colorimetric assay showed that the inner layer of galls contained a significantly larger amount of starch compared to the nodal regions, even when compared by unit weight (Fig. 8).

## Discussion

Weevil-induced galls were found only on the nodes in the shoots of the field dodder (Figs. 1 and S1). Many galls were found on the nodes of the shoots, and some galls were at the basal nodes of the inflorescences. Galls near the tips of the shoots were small, and those near the bases of the shoots were large. This indicates that galls are induced at the nodes near the tips of the shoots and grow during shoot or flower bud extension. Insect galls are generally induced at sites where cell division is actively proceeding^12,13^. The nodes at which axillary buds, consisting of actively dividing cells, are closely located, appear to be suitable for the weevil to form galls. The egg-laying behaviour of the weevil was observed only at the nodes near the shoot tips (Movie 1). This suggests that weevils somehow choose nodes as suitable places for gall formation and larval growth.

The volume of galls grew approximately 38.75-times by swelling of the nodal region. Under laboratory conditions, the largest galls were observed on the 13th day (Fig. 2e), which corresponded to the timing of the escape of weevils from the galls. The galls, from which the weevils escaped, stopped growing, gradually decreased in size, and withered (Fig. 2). The results suggest that the growth and maintenance of the galls are strongly affected by the presence of weevils inside the galls. Alternatively, the opposite possibility that weevils escaped from the galls because the physiological condition of the galls deteriorated cannot be ruled out. Further experiments are needed to test these hypotheses.

The galls observed in the laboratory (Fig. 2) tended to be smaller than the galls collected from the field (Figs. 1 and 3). We used the dodders parasitising the herbaceous plant *Nicotiana benthamiana* in the laboratory, while the galls were collected from dodders parasitizing the woody plant *Vitex rotundifolia* in the field*.* The host difference may have some effect on the physiological conditions of the dodder, and might also affect the development of galls. It is conceivable that various other environmental factors also affect the gall and weevil development, and need to be clarified in future studies.

The weevil larva inhabited the cavity at the centre of a gall (Fig. 1c,d). Despite the fact that larva actively fed the gall from the inside (Movie 3), the gall tissues remained thick (Fig. 1c). Fluorescence microscopy revealed that the inner cells were small, as found in callus tissues (Fig. 3c). These observations suggest that the inner cells show active cell division in response to larval ingestion. In the inner layer of the galls, vascular bundles were extensively developed (Fig. 4b). Extensive cell division and vascular development are general features of insect galls^14–17^.

*Cuscuta* plants, including the field dodder, are obligate stem holoparasites that acquire water and nutrients from host plants, except during the early germination stage^18^. Therefore, chlorophyll content and photosynthetic activity of the field dodder are usually very low^19–21^ as in other *Cuscuta* species^19,20,22,23^. In this study, we detected that *S. madaranus*-induced galls increased the photosynthetic activity in *C. campestris*. In mature galls, many chloroplasts were detected in the inner layer (Fig. 4b), whereas few chloroplasts were found in the outer layer (Fig. 4c), nodes, and shoots (Fig. S2). After the maturation of galls, the concentrations of Chl *a* and *b* in the inner layer of galls were approximately 2.8- and 3.0-times higher compared to that in the nodal regions, respectively (Fig. 5). In the whole gall, mean concentrations of Chl *a* and *b* were 1.6- and 1.8-times higher than those in the nodal regions. We may note that galls resulted from the nodal regions by enlarging the volume by approximately 38.75-times (see “Results” section). This means that gall formation by the weevils significantly increased the amount of chlorophyll resources in the field dodder. Analysis of CO_2_ absorption strongly suggested that galls remarkably increased CO_2_ assimilation and photosynthetic activity (Fig. 6). This study also shows that a large amount of starch accumulated especially in the inner layer of the gall (Figs. 7 and 8). Starch is stored in the chloroplasts^20,24^. Hence, it is reasonable that the inner layer with many chloroplasts is utilized as a food resource for *S. madaranus*.

In contrast to the inner layer of galls, the outer layer consisted of enlarged cells (Fig. 3d), and rarely contained vascular bundles and chloroplasts (Fig. 4c). The chlorophyll content in the outer layer of galls was not different from that in the stem (Fig. 5). Starch was not well accumulated (Figs. 7 and 8), and the feeding marks of the weevil larva did not exist in the outer layer (Figs. 1c, 3b, 4a, and 7). Hence, the outer layer of galls might not function as a food resource but rather function as a part of the thick wall of the gall, which could play important roles in defence from natural enemies, maintaining the internal gall environment in a fluctuating external environment, and might confer other features.

These results strongly suggest that the gall-inducing weevils have enhanced photosynthetic activity on the obligate parasitic plant, and modified the plant tissue to a nutrient-rich shelter for them. There is a possibility that starch accumulated in the galls originates not only from the photosynthetic products in the gall but also from those in the host plant. In the future, it is necessary to conduct a tracer experiment to test this possibility.

Plant gall formation is considered to be an extended phenotype of the insect^3^, and is an intriguing subject of research from various points of view, such as the ecological relevance of plant–insect interactions, mechanisms of manipulating plant morphogenesis, and their evolution. Thus far, several studies have been conducted to clarify the mechanisms of plant manipulations in insects. Phytohormones, namely auxins and cytokinins, have been suggested as key players in gall-formation^25–27^. In a few studies, it was clearly shown that indole-3-acetic acid is synthesized in insects^14,15,28,29^. Cytokinins have also been suggested to be synthesized in insects^14^ and, in some cases, are produced by the symbiotic bacterium, *Wolbachia*^30–33^. Some effector proteins secreted by insects are also candidates responsible for the formation of plant galls^34–36^. Because many gall systems are formed on trees^6,7^, it is difficult to maintain them in laboratories and to identify the mechanisms comprehensively through experimental manipulations. In this context, *S. madaranus* and *C. campestris* will be an excellent model system for studying insects-induced galls on plants, because they have several advantages, for example, they can be maintained and manipulated in the laboratory, the genome sequence of *C. campestris* is available^37^, *N. benthamiana* can be used as the host plant of *C. campestris*, allowing the use of various functional analysis tools^38,39^, and RNA interference generally works well for the weevils^40,41^. Because gall formation starts after the hole-digging and egg-laying behaviour of adult females (Movie 1), it is possible that some phytohormones and/or effector proteins sent with saliva and/or eggs are involved in the mechanisms as previously suggested^34,42^. Alternatively, substances produced by larvae developing in the galls may be involved in this phenomenon. To clarify these mechanisms, further studies using molecular techniques are needed.

To date, 121 species belonging to the genus *Smicronyx* have been reported^43^. This group includes both gall-forming and non-gall-forming species, and their phylogenetic relationships are largely unknown. The galls formed by some species belonging to the same genus, such as *S. jungermanniae*, *S. smreczynskii*, and *Smicronyx* sp., have been shown to increase at least a part of the photosynthetic apparatus^44–46^. This might imply that there are evolutionarily conserved molecular mechanisms among the *Smicronyx* species. In addition to the mechanistic studies, phylogenetic analyses and mapping of the gall-forming trait onto the phylogenetic tree will be needed for further understanding of the evolution of this group.

## Materials and methods

### Plants and insect

Broad bean (*Vicia faba*) and *N. benthamiana* were used as host plants in this study. Seeds of field dodder (*Cuscuta campestris*) were kindly provided by Professor Kazuhiko Nishitani (Tohoku University, Japan). Dodder seeds were scarified by soaking in 100% H_2_SO_4_ for 7 min, washed with distilled water, and sown in soil around the base of 1-week-old broad bean. The dodder was cultivated and allowed to parasitise the broad bean. Two weeks later, the dodder plants had grown from the infested site with multiple shoots, each growing to a length of 20 cm. Then, the tips of their shoots were cut off at 10 cm length and placed on the stems of 1-week-old broad beans or 4- to 6-week-old *N. benthamiana* to induce parasitism. At the beginning of each dark period, dodder parasitism was induced by infrared LED illumination (730 nm, 8 W/m^2^) for 15 min.

To establish the laboratory strain of the weevil, we collected the galls of *S. madaranus* from the field dodder parasitizing *V. rotundifolia* at Neagari, Nomi city, Ishikawa, Japan on 26 October, 2017. The weevils that emerged from the galls were reared on dodders parasitizing *N. benthamiana* in the laboratory. Under experimental conditions, only one individual was generally found in a gall. There were no apparent differences in the sizes of last instar larva, pupae and adult between the field-collected and laboratory-maintained weevils.

The laboratory strains of the dodder and weevils were used for several experiments as shown in the following sections. Other experiments were conducted using galls collected from *V. rotundifolia* in the field at Iwasekoshi-machi, Toyama city, Toyama, Japan on 23 July, 2019. Cultivation of all the plants, rearing of the weevils, and the experiments in this study were conducted at 28 °C in the long day regimen (14L10D).

### Observation of egg-laying and feeding behaviour of the weevil

Five adult weevils (mixed sexes) were set on the dodder parasitizing the broad bean in the laboratory. Time-lapse photography was performed with an 8-s interval for 5 days using a C270 HD Webcam (Logicool, Switzerland) connected to Mac mini (A1347, Apple). The interval shooting and creating the 15-fps time-lapse movie were conducted using shell script and Python 3 with an original source code. Feeding behaviour of larvae was observed and video was taken with a digital camera (Olympus Stylus TG-3 Tough) connected to a stereomicroscope (Leica M165 C) using a gall that was partially pierced with forceps.

### Observation of gall growth and escape timing of the weevil from the gall

Approximately 80 adult weevils (mixed sexes) of the strain were reared on the dodder parasitizing *N. benthamiana* in the rearing cage and allowed to induce the gall for 3–5 days in the laboratory. These adults were removed from the cage after confirming the formation of earliest galls with a width (length perpendicular to the shoot) of approximately 1.5 to 2 mm. These galls were then marked with markers. The gall widths were measured using a divider with a ruler. Observations were carried out daily until 3 days after the last weevil escaped from the gall. We analysed the galls harbouring only one larva, and the gall with multiple larvae were excluded from the analysis. Ten nodal regions with no galls were randomly chosen and cut near the shoot tips, and their images were immediately taken with a digital scanner (GT-X820, Epson) at a resolution of 600 dpi. Thereafter, stem widths at the nodes were calculated by image analysis with Fiji^47^. The volumes of the mature galls and nodal regions were estimated using the following formula:

$$ {\text{Vgall }} = 4/3\uppi \left( {{\text{Wgall}}/2} \right)^{3} , $$

$$ {\text{Vnode }} = \uppi \left( {{\text{Wnode}}/2} \right)^{2} {\text{WMgall}}, $$

where (Vgall) is the volume of the gall, (Wgall) is the width of the galls (mm) on the 13th day from the gall-forming, (Vnode) is the volume of the nodal regions, (Wnode) is the stem width at the nodes, and (WMgall) is the mean width of the galls (mm) on the 13th day from the gall formation.

### Gross and histological analyses of galls and shoots of the field dodder

This analysis was performed using mature galls (ca. 13–15 mm) collected from the field. Images of the dodder shoots and the appearance of galls were acquired with a digital camera (Leica DFC295) attached to a stereomicroscope (Leica M165 C). To observe the inside of the galls, they were cut using a razor blade, and the images were obtained as mentioned above. Sections of galls (200 µm in thickness) were cut using a LinearSlicer PRO7 (Dosaka EM, Kyoto, Japan). Sections of the shoots (ca. 500 µm) were made with a razor blade. Cell walls were stained with Calcofluor White ST (American Cyanamid Co.). Thereafter, the cell sizes of the galls were observed in the sections using a confocal laser scanning microscope (LSM 5 PASCAL, Carl Zeiss) with an emission at 420–480 nm after excitation at 405 nm. The distribution of chloroplasts in the whole gall or shoots was observed with an AZ 100 M microscope (Nikon) using chlorophyll autofluorescence with an emission at 600–660 nm after excitation at 540–580 nm, and photographed with a DS-Fi3 camera (Nikon). Detailed images of the chloroplasts and vascular bundles were observed using chlorophyll and lignin autofluorescence, respectively, with a BX-50 FLA microscope (Olympus) using the same emission and excitation profiles—an emission wavelength longer than 420 nm after excitation at 330–385 nm—and photographed with a Coolsnap cf camera (Nippon Roper). To detect starch on the sections of galls and shoots, the sections were treated with 1% Lugol’s iodine solution at 25 °C for 5 min in 2 mL tubes with gentle shaking as described^48^. Images of the treated sections were acquired with DFC295 connected to M165 C.

### Quantification of starch in galls and nodal regions

The mature gall (approximately 7–10 mm) induced in the laboratory was cut and divided into the inner and outer tissues with a razor blade, and 10 mg fresh weight of each part of the gall was used as one sample. Ten milligrams fresh weight of a nodal region, including 5 mm of stems at the node, was considered as one sample. Starch measurements were performed by colorimetric assay using a Starch Assay Kit (Cell Biolabs, USA), according to the manufacturer’s instructions. Each sample of the three regions was analysed in duplicate, with one control well per sample well. Absorbance was measured at 540 nm using microplate reader (Synergy HTX, BioTek). The amount of starch per unit weight (g) was calculated for gall inner layers, outer layer, and nodal regions separately.

### Estimation of chlorophyll concentration

Extraction of Chl and estimation of their concentrations were done as described by Porra et al.^49^ with minor modifications. This analysis was performed using a laboratory dodder strain. The mature gall (approximately 7–10 mm) was cut and divided into the inner and outer tissues with a razor blade, and each part of the gall was used as one sample. Approximately 3 cm long stem of the dodder was counted as one sample. Three nodal regions, including 5 mm of stems from the node, were pooled as one sample because of the small amounts. Twelve samples per part were weighed and used for extraction of Chl *a* and *b* using 80% acetone buffered with 2.5 mM sodium phosphate (pH 7.8). The absorbance of chlorophylls was measured using NanoDrop OneC (Thermo Fisher Scientific Inc.). The concentration of Chl *a* and *b* was calculated using the following formula^49^ and standardized to fresh weight of the sample.

$$ {\text{Chl}}a\left( {\upmu {\text{g}}/{\text{mL}}} \right){\text{ }} = 12.25 \times {\text{A}}^{{663.6}}  - 2.55 \times {\text{A}}^{{646.6}} $$

$$ {\text{Chl}}b\left( {\upmu {\text{g}}/{\text{mL}}} \right){\text{ }} = 20.31 \times {\text{A}}^{{646.6}}  - 4.91 \times {\text{A}}^{{663.6}} $$

### Quantification of CO_2_ absorbance

Using shoots and mature galls induced in the laboratory, the photosynthetic rate was measured by the gas exchange method with a laboratory-constructed system as described previously^50^ with minor modifications. In short, approximately 3–5 g of galls (10 galls) or 2–3 g of shoots (50 shoots cut to a length of ca. 7 cm) were pooled into a single sample, and placed in an acrylic chamber (12 × 10 × 1.5 cm, W × D × H). The CO_2_ concentration in the air entering and leaving the chamber was measured with an infrared CO_2_/H_2_O gas analyser (LI-7000; Li-Cor Inc., Lincoln, USA). The CO_2_ concentration in the air leaving the chamber was set at 400 µmol mol^−1^. The photosynthesis measurements were performed after 10–15 min of acclimation. The flow rate was 300–350 ml min^−1^ that was adjusted using a flow meter, and humidity of the air was regulated at 0.5–2.5 kPa using a dew point generator (LI-610, Li-Cor, NE, USA). The measurements were conducted under both dark and light-saturated conditions using the same sample, 0 and 1100 µmol m^−2^ s^−1^ of photosynthetic photon flux density, at 25 °C for calculating the photosynthetic rate. Photosynthesis rates was calculated as the difference of CO_2_ concentration between dark and light-saturated conditions as described^50^. Five gall samples and five shoot samples were used for photosynthesis measurements. To obtain the total area and length of galls or shoots, the measured galls and shoots were scanned with a digital scanner (GT-X820, Epson) at 300 dpi resolution. The total area or total length of each sample was calculated by image analysis using R^51^ v3.3.3 and EBImage package in Bioconductor (URL https://www.bioconductor.org/packages/release/bioc/html/EBImage.html). The total lengths were approximated by dividing the perimeter of each sample by two or π for the shoot or gall, respectively. The amount of CO_2_ absorption per unit area (cm^2^) or unit length (cm) was calculated for the galls and shoots, respectively.

### Statistical analysis

Statistical analyses were performed using the R package^51^ ver. 4. 0. 2. The Wilcoxon rank sum test with Bonferroni correction was used for the starch content and the chlorophyll concentration data. Welch’s t-test was used for the analysis of the CO_2_ absorption data.

## Supplementary Information

Supplementary Information 1.

Supplementary Video S1.

Supplementary Video S2.

Supplementary Video S3.

## Data Availability

This article has no additional data.
