# Supplementary material for: A new galling insect model enhances photosynthetic activity in an obligate holoparasitic plant
Source: Sci Rep. 2021 Jun 21;11:13013. doi: 10.1038/s41598-021-92417-3 (PMC8217554; doi:10.1038/s41598-021-92417-3)
Supplement: Supplementary file 1 — Supplementary Information 1. [file 41598_2021_92417_MOESM1_ESM.pdf]

Supplementary Material for:

A new galling insect model enhances photosynthetic activity in an obligate holoparasitic plant

Ryo Murakami[a], Ryo Ushima[a], Ryoma Sugimoto[a], Daisuke Tamaoki[b], Ichirou Karahara[b], Yuko Hanba[c], Tatsuya Wakasugi[b], and Tsutomu Tsuchida[b*]

[a] Graduate School of Science and Engineering for Education, University of Toyama, Toyama City, Toyama 930-8555, Japan

[b] Faculty of Science, Academic Assembly, University of Toyama, 3190 Gofuku, Toyama City, Toyama 930-8555, Japan

[c] Faculty of Applied Biology, Kyoto Institute of Technology, Matsugasaki, Sakyo-ku, Kyoto 606-8585, Japan

*Corresponding author:

Tsutomu Tsuchida

tsuchida@sci.u-toyama.ac.jp

+81-76-445-6553

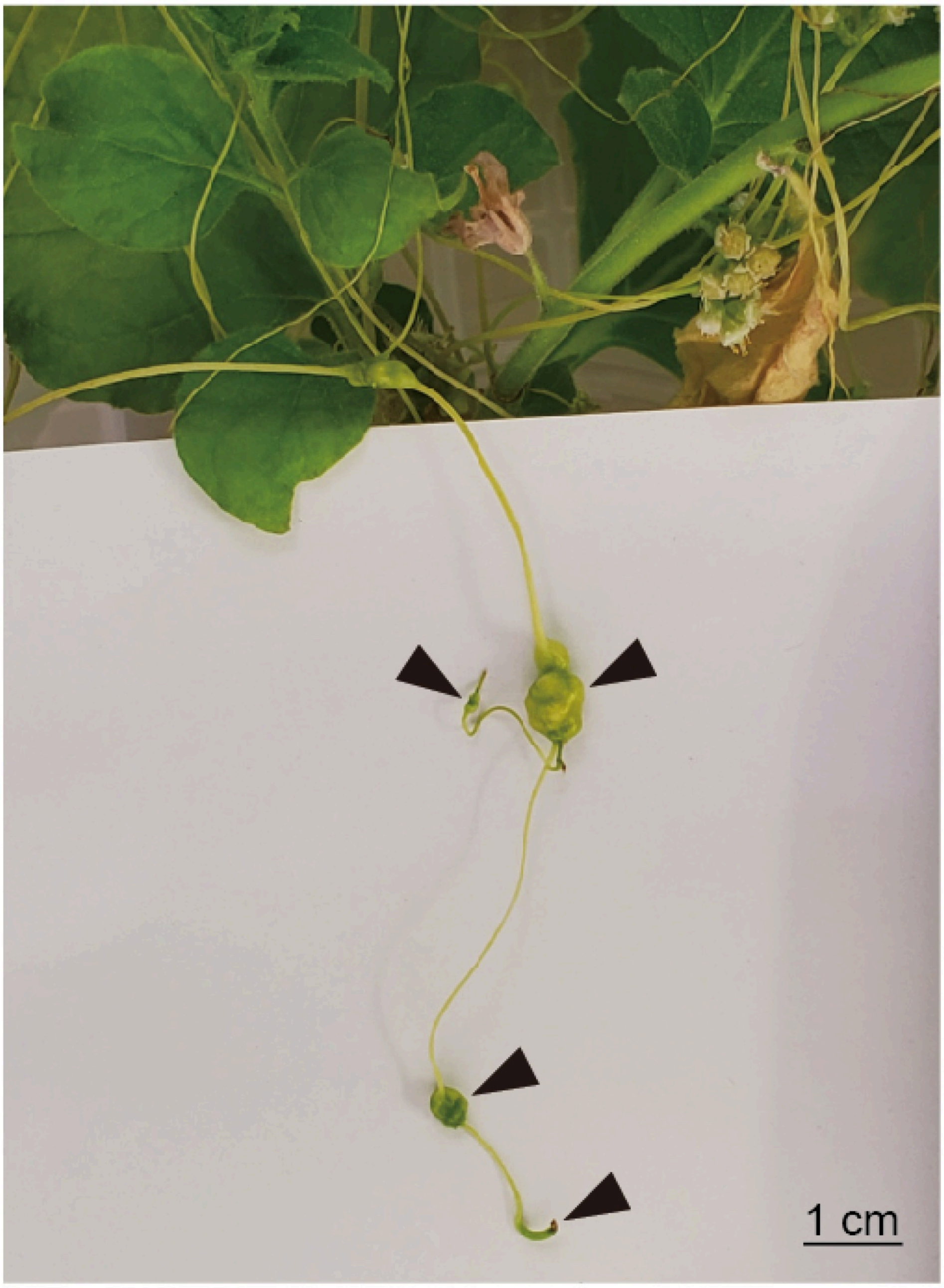

Fig. S1 Galls of *Smicronyx madaranus* formed in the nodes of *Cuscuta campestris* in the laboratory. Arrow head, gall.

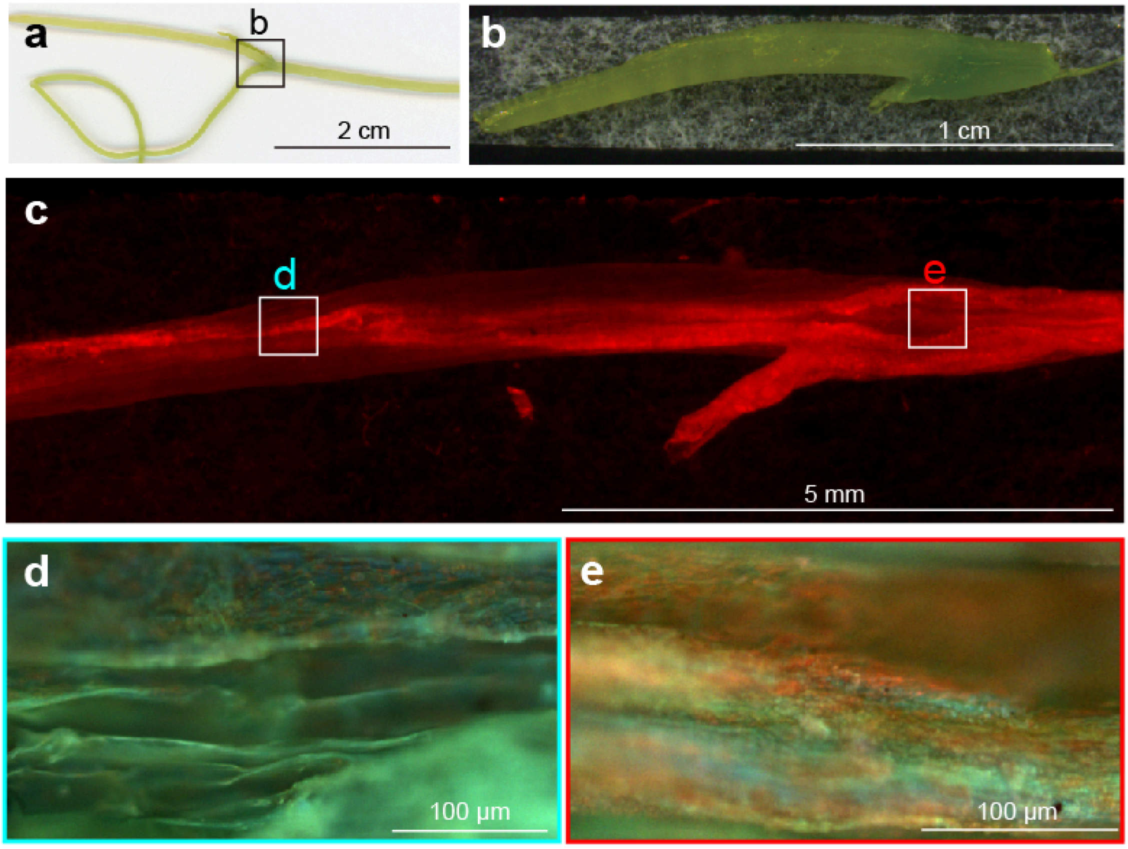

Fig. S2 Distribution of chlorophylls and chloroplasts in the shoot of *Cuscuta campestris*. (a) Shoots of *C.campestris*, and (b) sagittal section of the branching point that corresponds to b in panel a. (c-e) autofluorescence of chlorophylls (red) and lignin (green), (d) and (e) magnified images of the points in shoot and the branching point in panel c.

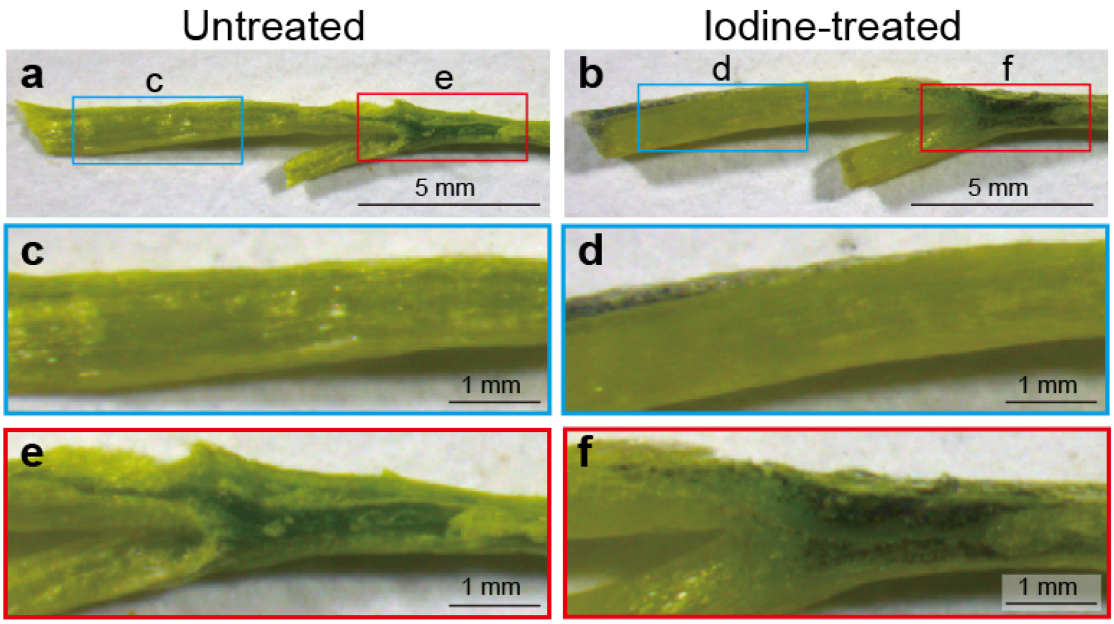

Fig. S3 Distribution of starch in the shoot (a, c, and e) Sagittal section of the shoot and branching point. In (b, d, and f), starch was detected as blue-black colour upon staining with Lugol's iodine solution.

Supplementary videos

Movie S1 Egg-laying behaviour of *Smicronyx madaranus* (left). Enlarged image around the weevil (right).

Movie S2 Plant-boring behaviour of *Smicronyx madaranus* (left). Enlarged image around the weevil (right).

Movie S3 A larva feeding the inside tissue at the mid stage of the gall.
